# The Effect of P4 + eCG Estrus Induction Protocol during the Deep and the Transition Anestrous Period on the Reproductive Performance of Crossbred Dairy Goats

**DOI:** 10.3390/biology9100311

**Published:** 2020-09-24

**Authors:** Francisco G. Véliz-Deras, César A. Meza-Herrera, Miguel Mellado, Viridiana Contreras-Villarreal, Leticia R. Gaytán-Alemán, Juan M. Guillén-Muñoz

**Affiliations:** 1 Unidad Laguna, Universidad Autónoma Agraria Antonio Narro, Periférico Raúl López Sánchez y Carretera a Santa Fe, Torreón 27054, Coahuila, Mexico; velizderas@gmail.com (F.G.V.-D.); mmellbosq@yahoo.com (M.M.); dra.viridianac@gmail.com (V.C.-V.); zukygay_7@hotmail.com (L.R.G.-A.); 2 Unidad Regional Universitaria de Zonas Áridas, Universidad Autónoma Chapingo, Bermejillo, Durango 35230, Mexico; cmeza2020@hotmail.com

**Keywords:** seasonal anestrous, estrus induction, ovulation rate, fertility rate, pregnancy rate

## Abstract

**Simple Summary:**

The effect of an ultra-short progesterone (P4) plus equine chorionic gonadotropin (eCG) based estrus induction protocol during deep seasonal anestrous, or the reproductive transition period upon reproductive performance in an arid environment (26° N) was assessed. Results confirm a multidimensional response of goats regarding the effectiveness of P4 + eCG estrus induction protocols, mainly modulated by a specific time within the anestrous season (June) or even by specific management or a particular environment at herd level (herd 1 in this study), although, it is quite remarkably non–dependent on the animal’s body weight or body condition score.

**Abstract:**

Seasonal reproduction restricts the offering of goat commodities across the year. Therefore, it is crucial to improve diverse strategies to induce the reproductive response in goats during the anestrus stage. The effectiveness of a short P4 + eCG-based estrus induction protocol during both the deep anestrous (March) or the reproductive transition period (June) upon the reproductive performance of crossbred dairy goats was assessed. Adult, anestrous, 24–30-month- old dairy crossbred (Saanen–Alpine–Nubian × Criollo) goats (*n* = 123) from two commercial herds and 10 sexually active goat bucks were used. Before the trials, the anestrous status of goats was confirmed. Then, goats were randomly allocated into two different experiments. In Exp. 1, we tested the effect of different doses (D) of intramuscular progesterone (P4; 10 or 20 mg + eCG (100 UI)) and type of breeding (TB), natural mating (NM), or artificial insemination (AI), on two commercial goat herds (H1 & H2), in March (deep anestrous). In Exp. 2, we evaluated the effect of D (P4; 10 or 20 mg + 100 UI eCG) in goats subjected to NM, and either during deep anestrous (March; M) or transitional anestrous (June; J), in two commercial herds. After breeding, conception and pregnancy were diagnosed with ultrasound scanning. The response variables were estrus induction (EI; %), estrus latency (EL; h), ovulation (OVP; %), ovulation rate (OR; units), fertility (FERT; %), and pregnancy (PREG; %). No differences (*p* > 0.05) in live weight (LW) and body condition score (BCS) occurred between herds in both trials. In Exp. 1, EI, EL, OVP, OR, FERT, and PREG were affected (*p* < 0.05) by the H–TB–D interaction, whereas in H1 + P4–20 combination had the highest (*p* < 0.05) EI, EL, and OVP values. Irrespective of TB, H1 had the largest (*p* < 0.05) OR, independently of TB or D. Also, the lowest (*p* < 0.05) OVP occurred in the AI + P4–10 group, while the AI had the lowest (*p* < 0.05) FERT, irrespective of D. FERT and PREG were two-fold higher (*p* < 0.05) in NM compared with AI. In Exp. 2, EI, EL, OVP, OR, FERT, and PREG were affected (*p* < 0.05) by the H–M–D interaction. In general, H2 + P4–10 had the lowest (*p* < 0.05) reproductive outcomes in March, whereas H1 had the largest (*p* < 0.05) values in either month. No differences (*p* > 0.05) between P4 doses occurred for EI, OVP, OR, FERT, and PREG. Yet, the largest (*p* < 0.05) EL occurred with P4–20 in June. No correlations (*p* > 0.05) occurred between LW and all the reproductive variables. BCS was positively correlated (*p* < 0.05) with EI (0.34), OVP (0.44), OR (0.58), and PREG (0.20). Also, positive correlations (*p* < 0.05) occurred between EI with EL (0.83), OVP (0.80), OR (0.64), and PREG (0.56); EL with OVP (0.58), OR (0.44), and PREG (0.42); OVP with OR (0.79) and PREG (0.70), as well as OR and PREG (0.63). Results of these studies confirm a multidimensional response regarding the effectiveness of P4 + eCG for estrus induction in goats mainly modulated by a specific time within the anestrous season, or even by specific management or a particular environment at the herd level (H1), although quite remarkably independent of the animal’s LW or BCS at herd level. Moreover, the best reproductive outcomes occurred with NM in June. The most reproductive variables were similar using either 10 or 20 mg P4 + 100 IU eCG, giving the possibility to lessen the scale in the use of exogenous hormones while obtaining acceptable out of season reproductive response.

## 1. Introduction

Reproductive seasonality is an adaptation mechanism developed in response to environmental changes and food availability across a given year. Estrus and ovulation synchronization in small ruminants can be controlled by different means, such as the administration of progesterone (P4) plus equine chorionic gonadotropin (eCG), to counteract reproduction seasonality [1], using either artificial insemination or natural mating [2]. Such a reproductive seasonality occurs in temperate regions of the world and negatively affects products for marketing of sheep and goats [3,4], from regions ≥25° N [5,6] to the higher latitudes >40° N and S; [7]. Fluorogestone acetate (FGA; doses between 20 and 40 mg) [8], medroxyprogesterone acetate (MAP; doses of 60 mg) [9], and the controlled internal drug release (CIDR, natural P4 0.3 g) [10] are commercially available hormones commonly used in small ruminants. All of these are applied intravaginally from five to 14 days [11,12]. At the moment of withdrawal and to induce follicular growth and ovulation in acyclic females, eCG in doses from 100 to 600 IU is also used [13]. Short-term treatments with high P4 levels control follicular dynamics and, consequently, improve conception rate [14]. In goats, P4 + eCG short–term treatment was effective at inducing estrus and improving pregnancy rate during the seasonal anestrous [15].

Most P4 analogs are usually expensive and, generally, inaccessible to most producers from different regions of the world. The advantages of ultra-short treatments are, on one hand, the prevention of vaginitis, as reported with vaginal sponges [16], as well as that they contain natural progesterone, unlike analogs. Therefore, the use of reduced hormonal doses and simplified methods of administration to induce estrus in goats, testing ultra–short, single administration of natural P4 + eCG has been a sensible welfare issue [1,17]. While the eCG dose can be considered already low, we hypothesized that the traditional intramuscular dose of P4 (20 mg) can be further decreased when applied either in deep anestrous or during the anestrous transition period without compromising the goat reproductive response. This study aimed to evaluate the reproductive performance of anestrous adult crossbred dairy goats treated with two different intramuscular doses of natural P4 (10 or 20 mg) + eCG (100 IU) during both the deep (March) and the transition (June) anestrous period in an arid environment (25° N). Moreover, the effect of natural mating (NM) or artificial insemination (AI) as well as the relationships among live weight (LW) and body condition score (BCS) with reproductive variables were also tested.

## 2. Materials and Methods

### 2.1. General

All the experimental procedures, methods, and managing of the trial experimental units used in this study comply with the guidelines for the ethical use, care, and welfare of animals in research at international [18] and national [19] levels, with institutional approval reference number UAAAN–UL–18–3043.

### 2.2. Location, Environmental Conditions, Animals, and their Management

This study was conducted in two commercial dairy goat farms (H1 and H2) during both the seasonal anestrous (March) and the reproductive transition period (June) in northern Mexico (26°23′ N; 104°47′ W). All goats had water and alfalfa hay ad libitum (17% crude protein; CP, and 1.95 Mcal of metabolizable energy; ME) and each animal received 200 g of a commercial concentrate (14% CP and 2.7 Mcal of ME). Adult, anestrous, dairy-type goats (*n* = 123, Saanen–Alpine–Nubian × Criollo, 24–30-months old) and 10 sexually active bucks of proven fertility were used. Goats had a BCS of 2.5 on a scale of 0–5 (0 = very thin and 5 = very fat, with increments of 0.25 [20]), with an average LW of 43.7 ± 1.4 kg, and were randomly allocated into two different experiments.

### 2.3. Experiment 1: Effect of Different Doses of Intramuscular Natural Progesterone (10 or 20 mg) + eCG, and Either Natural Mating or Artificial Insemination in Two Herds on Reproductive Performance of Anestrous Goats

**Figure 1:**
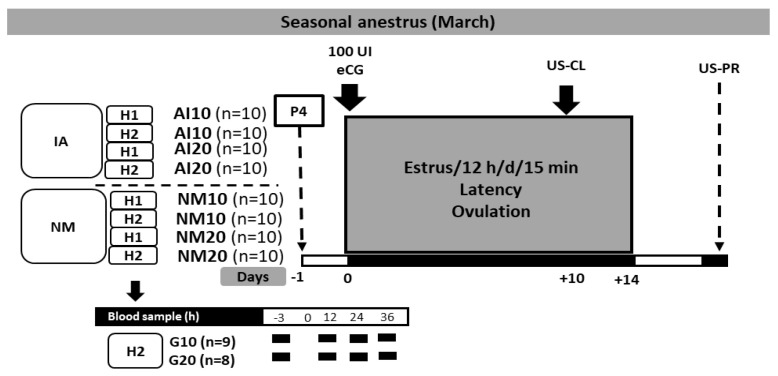
Experimental design of the ultra–short protocol based on P4 + eCG in crossbred adult anestrous goats (Saanen-Alpine-Nubian x Criollo, *n* = 80). *Note:* H1 = herd 1; H2 = herd 2, P4 = doses of intramuscular P4 either 10 or 20 mg goat^−1^; goats treated with P4 and exposed to either natural mating (NM10 or NM20) or artificial insemination (AI10 or AI20). All goats were treated with 100 IU eCG, 24 h after P4–administration; (day 0). US–CL = ultrasound to determine the presence of corpus luteums 7 d after the estrus peak. US–PS = ultrasound to determine pregnancy 45 d after breeding. Serum P4 concentration was quantified four times; during the first hour after eCG administration and 12, 24, 36 h later.

During the pre–trial stage, the anestrous status of all goats (*n* = 80) was confirmed using two transrectal ultrasound scans (mid-February), using a 7.5 MHz human prostate transducer (Aloka 500, MHz linear array; Corometrics Medical Systems, Inc., Wallingford, CT, USA). The transducer was lubricated and then introduced into the goat’s rectum to define the type of ovarian structures observed in both ovaries. Those goats with corpus luteum were not further considered in the study. Thereafter, on March 1, four homogeneous groups (*n* = 20, each) were formed regarding LW and BCS. Goats were administered either 10 or 20 mg P4 (Progesvit, Brovel, Mexico, DF). Then, goats were exposed to either natural mating (NM10 or NM20) or artificial insemination (AI10 or AI20). All goats were treated with 100 IU of eCG (Ceva Sante Animale, Libourne, Francia), 24 h after the administration of natural P4 (day 0). A third ultrasonographic scanning (US–CL) was performed to determine the presence of corpus luteum on d 10 after eCG administration. Thereafter, a fourth US–PR was performed to evaluate pregnancy status 45 d after breeding. Four blood samplings were collected to quantify serum P4 concentrations, during the first hour after eCG administration and 12, 24, 36 h later. The time of the P4 administration was defined as hour “0”. A schematic representation of the main activities performed during the whole experimental period is depicted in Figure 1.

### 2.4. Experiment 2: Effect of Different Doses of Intramuscular Natural Progesterone (10 or 20 mg) + eCG, Month of the Anestrous Season (March or June), and Herd (H1, H2), in Goats Exposed to Natural Matingt, on Reproductive Performance of Anestrous Goats

**Figure 2:**
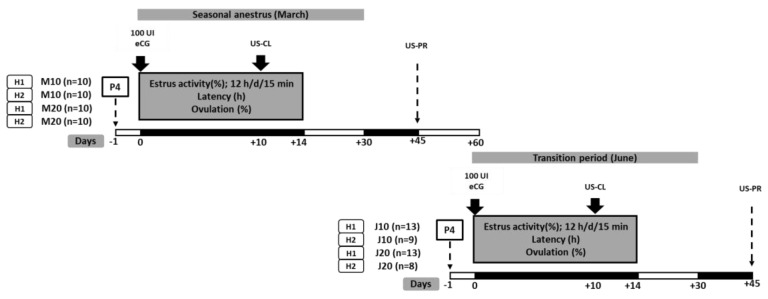
Experimental design of the ultra–short estrus synchronization protocol based on progesterone in crossbred (Saanen-Alpine-Nubian x Criollo) anestrous goats according to the month of the anestrous season. *Note:* H1 = herd 1; and H2 = herd 2. P4 = intramuscular injection of natural progesterone. M10 and M20, 10 and 20 mg of P4 were administered, respectively (at the beginning of seasonal anestrous); J10 and J20, 10, and 20 mg of P4 were administered, respectively (during the transition period to the reproductive season). Exogenous progesterone was administered intramuscularly. All females were treated with 100 IU eCG (Ceva SanteAnimale, Libourne, France), 24 h after P4–administration (day 0). While an US–CL = an ultrasound scanning was performed to determine the presence of a corpus luteum at d 7 after the estrus peak, an US–PR = pregnancy was evaluated at 45 d after buck joining.

Before the June trial, the anestrus condition of goats (*n* = 43) was assessed using two transrectal (one per week) ultrasound scans (US). US were performed as previously explained in Experiment 1. From the results obtained in March regarding NM, we compare the effect of the two P4 doses (10 or 20 mg), the month within the anestrous season (deep anestrus, March vs. transitional anestrus, June) in the same two commercial herds, yet, with a new set of goats for the experiment of June. Therefore, on June 16, four groups of goats were conformed (*n* = 13, 9, 13, 8) as shown in Figure 2. Goats were homogeneous in terms of LW and BCS. Also, four sexually active bucks (BCS 3.0 ± 0.4 units, and 2–3-yr-old) of proven fertility were used. Two aproned bucks were used for detecting estrus as well as for NM; the other two bucks provided semen for AI. Ejaculates were collected with an artificial vagina; viability, concentration, and motility were recorded. Thereafter, 200 × 10^6^ spermatozoa per dose were used to inseminate does. The diluted fresh semen was kept at 35 °C and used 20 min after collection by a previously outlined procedure [6]. The AI10 and AI20 goats were inseminated transcervically 12 and 24 h after the onset of estrus. Goats from the NM10 and NM20 in estrus were breed by the two sexually active bucks.

To quantify serum P4 concentration, two groups of goats from AI or NM were randomly assigned to receive 10 or 20 IU eCG. Blood samples (10 mL) were collected prior to the morning feeding, (15 min × 6 h) by jugular venipuncture into sterile vacuum tubes (Corvac; Kendall Health Care, St. Louis, MO, USA) and allowed to clot at room temperature for 30 min. Serum was separated by centrifugation (1500× *g* for 15 min), decanted, and transferred to polypropylene microtubes (Axygen Scientific, Union City, CA, USA) to determine the P4–release curve: 3 h prior P4 administration, and then every 12 h (i.e., 12, 24, and 36 h); samples were stored at −20 °C until hormonal analysis. Serum P4 concentration was measured in duplicate throughout a direct solid–phase RIA (Coat–a–Count, DPC, Los Angeles, CA, USA). Samples and calipers were P4–marked as ^125^I, after incubation, the tube’s contents were vacuumed and the radioactive unit was sampled. The calibration curve was established and the unknown values were determined by curve interpolation (IMMUNOTECH, 2015). The analytical sensibility of the kit is of 0.03 ng/mL, with an intra-test variation coefficient of 4.76%, for the control of the kit 1474 ng mL^−1^ (Lot P105 0.90–1.57 (1.20–0.30 ng mL^−1^). The main activities performed during the second experiment are shown in Figure 2.

### 2.5. Estrus Induction, Estrus Latency, Ovulation Percentage, and Pregnancy Rate

Before the study, two ultrasounds were performed to determine if goats were anestrous. This was done 14 and 7 days before the treatment. Since day 1 of mating, the onset of the estrus induction was evaluated twice daily (7:00 and 19:00 h; 15 min each) every 12 h during 15 consecutive days; thereafter, teaser bucks were removed from the goats’ pen. Also, estrus latency was evaluated from the time of eCG administration until the manifestation of estrus [21]. Later on, two ultrasound (US) scans were performed as described previously. The third US was performed to determine ovulation rate; the second to determine pregnancy rate. On d 10 after eCG administration, the number of corpus luteum was registered and the fourth US was performed to detect the presence of embryonic structures on d 45 after buck introduction. Ovaries were visualized at an image magnification of 1.5×, and the number and diameters of the corpus luteum in each ovary were recorded as previously described [4]. Corpus luteum was identified on gray scale as a hypoechoic area, whereas pregnancy was visualized by the presence of a perfectly elongated embryo implanted in a hypoechogenic endometrium with at least four delimited layers (>8–10 mm) with the existence of sub-endometrial vascular flow and observable myometrial contractions. Ultrasonographic images were also recorded for retrospective analyses. All US were performed by the same well–trained operator. During the experimental period, the same personnel and the same procedures were used in the places where the study was carried out. The response variables included estrus induction (EI, %), estrus latency (EL, h), ovulation (OVP, %), ovulation rate (OR, units), fertility (FERT, %), and pregnancy (PREG, %).

### 2.6. Statistical Analyses

The response variables were analyzed using a CRD–ANOVA. The model included the response variables as affected by hormonal treatment (P4 + eCG), dose (10 or 20 mg), season (March or June), type of breeding (NM or AI), and herd (Herd 1 & 2), as well as first-order interactions. These analyses were carried out with PROC GLM of SAS (SAS Institute Inc, Cary, NC, USA). Serum P4 concentration across time was assessed for normality using the UNIVARIATE procedure. Log^10^ transformation was necessary before analysis to overcome the skewness of P4 data. The models included the treatment effect in the main plot, which was tested using the animal within treatment as the error term. Both time and treatment x time interaction were included in the subplot and tested by using the residual mean square (PROC MIXED of SAS). Significant differences were further investigated using the PDIFF option. Treatment was examined within sampling time when treatment x time interaction occurred [22]. To quantify the association between two response variables, Pearson correlations were carried out. Spearman´s correlation coefficient was used to evaluate qualitative non-normally distributed, non-parametric variables, i.e., BCS [23]. The significance level was set at *p* < 0.05.

## 3. Results

### 3.1. Experiment 1

**Figure 3:**
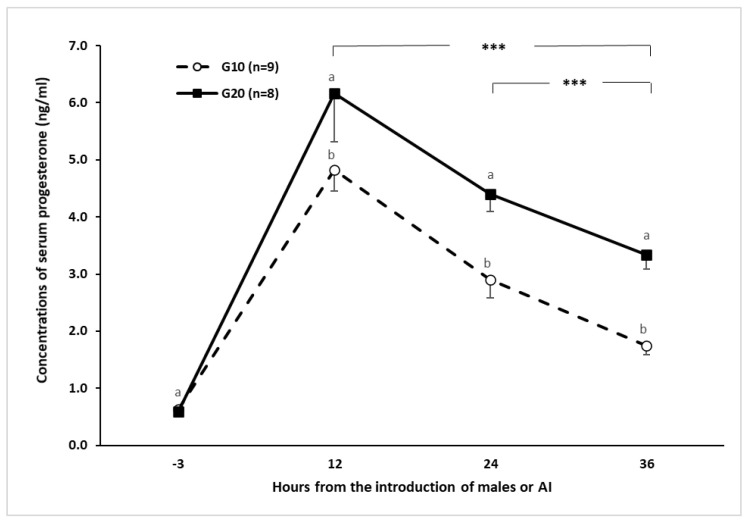
Mean serum progesterone concentration in goats treated with 10 or 20 mg of progesterone at −3, 12, 24, and 36 h from buck introduction in adult anestrous crossbred (Saanen-Alpine-Nubian x Criollo) goats subjected to an ultra–short P4–eCG based estrus induction protocol during the seasonal anestrous (March; 25° N). ^a, b^ = (*p* < 0.005); *** = (*p* < 0.001).

To confirm the anestrous status of goats, additional to the pre–trial transrectal US scanning, blood samples were collected to quantify serum P4 concentrations to a sample of animals from each group (eight and nine goats). Serum P4 levels lower than 1 ng/mL for the pre–trial period were considered in anestrous, reaffirming the absence of luteal activity, follicular development, and ovulation. No differences (*p* < 0.05) between herds occurred regarding LW (H1 = 43.6 ± 1.3 & H2 = 43.6 ± 1.3) and BCS (H1 = 2.1 ± 0.05 & H2 = 2.1 ± 0.05). Thereafter, upon P4 administration, goats receiving 10 or 20 mg P4 showed a similar trend, reaching their highest serum P4 level (*p* < 0.05; >4 ng/mL) 12 h after P4–administration, afterward serum P4 concentration decreased. Nonetheless, serum P4 concentrations were always higher (*p* < 0.05) in the G20 group along the sampling period (Figure 3).

The reproductive response according to the P4 doses type of breeding and herd is shown in Table 1. The highest EI and OR were higher (*p* < 0.05) in the H1. No differences (*p* > 0.05) between herds occurred for EL (49.8 h), OVP (77.5%), FERT (39.7%) and PREG (31.2%). Regarding P4 dose, the highest (*p* < 0.05) values for both EI (82.5 vs. 60%) and EL (56.3 vs. 43.5 h) was higher for the 20 mg P4, with no differences between P4 doses for OVP (60%), OR (1.55 units), FERT (39.5%) and PREG (31%). Interestingly, however, EI, EL, OVP, OR, FERT, and PREG were affected (*p* < 0.05) by the interaction herd x type of breeding x dose. In general, both H1 and P4–20 mg depicted the higher values (*p* < 0.05) regarding EI, EL, and OVP, irrespective of type of breeding, the largest (*p* < 0.05) OR (n) occurred in the H1 irrespective of type of breeding or P4 dose. Besides, the lowest (*p* < 0.05) OVP occurred in the AI + 10 mg group, while the lowest (*p* < 0.05) FERT occurred in the AI group, irrespective of P4 dose. Finally, both FERT and PREG were affected (*p* < 0.05) by type of breeding with values two–fold higher for NM compared to AI.

### 3.2. Experiment 2

**Figure 4:**
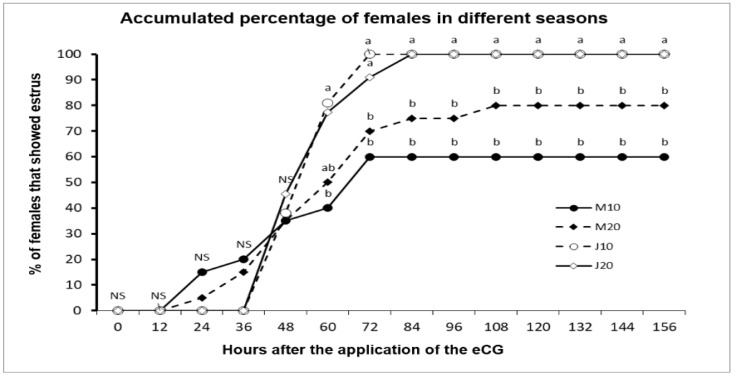
Accumulated percentage of estrus activity in adult crossbred (Saanen-Alpine-Nubian x Criollo) goats subjected to an ultra–short P4 (10 or 20 mg) & eCG based estrus induction protocol during the seasonal anestrous (March = M10 or M20) or the transition reproductive period (June = J10 or J20) in Mexico (25° N). (Note: a, b = Values with different superscripts differ (*p* < 0.05).

Reproductive variables were affected by season of the year. P4 doses in two commercial herds with crossbred adult goats during the anestrous season are presented in Table 2. All goats were confirmed in anestrous during the pre–trial period. No herd differences (*p* > 0.05) occurred in either LW (H1 = 43.4 ± 1.1; H2 = 44.6 ± 1.1 kg) or BCS (H1 = 2.1 ± 0.06; H2 = 2.6 ± 0.06 units). EI, EL, OVP, OR, and PREG were higher (*p* < 0.05) in herd 1 compared to herd 2. Only FERT was higher (*p* < 0.05) in herd 2 compared to herd 1. Also, no differences (*p* > 0.05) regarding EI, OVP, OR, FERT, and PREG occurred between P4 doses. An increased (*p* < 0.05) EL was observed in the group receiving 20 mg P4. Moreover, the highest (*p* < 0.05) values for EI and EL occurred in June compared to March. Nonetheless, all variables were affected (*p* < 0.05) by the herd x month of breeding x P4 dose interaction. In general, herd 2 showed the lowest (*p* < 0.05) reproductive outcomes when P4 was 10 mg in March. In general, herd 1 showed a better (*p* < 0.05) reproductive outcome. The accumulated percentage of goats showing estrus according to P4–dose in both March and June are shown in Figure 4. No differences (*p* > 0.05) during the first 60 h between the J10 vs. M10 groups (>80% & 40%, respectively; *p* > 0.05) occurred. Besides, no differences between P4 doses (*p* > 0.05) were found, except for EL. Besides, EL differed (*p* < 0.001) between March to June (37.2 ± 3.44 h vs. 58.0 ± 3.32 h).

Reproductive responses affected by the interactions between month of breeding–herd or month of breeding–P4 dose are shown in Table 3. Although herd 1 depicted the highest (*p* < 0.05) reproductive outcomes in March, no herd differences (H1 vs. H2; *p* > 0.05) were observed in June regarding EI, EL, OVP, OR, and PREG. Moreover, a month of breeding–P4 dose interaction was observed for EI and EL, observing, in general, the best results (*p* < 0.05) in June, irrespectively of P4 dose, with the lowest values for breeding in March + 10 mg P4.

Correlation coefficients among LW, BCS, and the reproductive variables are shown in Table 4. Unexpectedly, LW was not related (*p* > 0.05) with any reproductive variable, but BCS was positively associated (*p* < 0.05) with EI, OVP, OR, and PREG. Further, EI was positively related (*p* < 0.05) with EL, OVP, OR, and PREG. A medium relationship (*p* < 0.05) was also observed with EL and OVP, and OR, and PREG. The same was true (*p* < 0.05) for OVP, and OR and PREG, as well as between OR and PREG (*p* < 0.05).

## 4. Discussion

We hypothesized that the traditional intramuscular dose of P4 (20 mg) can be reduced when applied either in deep anestrous (March) or during the anestrous transition period (June) without compromising the goat reproductive response. Moreover, we also tested the use of NM or AI as well as the potential relationships among LW, BCS on reproductive variables. Results support our working hypothesis in that, except for estrus latency, all other reproductive variables were similar irrespective of the P4 dose. These results also define an interesting multifactorial reproductive response regarding the effectiveness in the use of the estrus induction protocols in that it was modulated by a specific time within the anestrous season (i.e., June), specific management, or a particular environment at herd level (i.e., herd 1), as well as according to the type of breeding. Moreover, and quite remarkable, such reproductive responses were observed with no differences in LW or BCS between herds. The correlation analyses established that the reproductive response variables were not correlated with LW although positively related to BCS.

The administration of exogenous P4 (10 or 20 mg) + 100 IU eCG 24 h after P4 was effective at inducing the estrus activity of goats during the anestrous season. Both P4 doses reached their highest serum P4 level 12 h after eCG administration, surpassing the 6 ng mL^−1^ when using 20 mg P4, which is similar to the result with the use of a natural P4 CIDR protocol [24]. Nonetheless, in our study, serum P4 levels were still high (>2 ng mL^−1^) for at least two days after P4 administration. At the beginning of the reproductive season, a single administration of 20 mg P4 is enough to eliminate the infertile cycles (short cycles), and it allows the first estrus to be accompanied by ovarian activity and fertility [25]. Short cycles have been associated with shorter luteal phases, a smaller corpus luteum, and lower circulating progesterone concentrations [26]. Besides, serum P4 levels have been inversely related to the size of the dominant follicle and follicular turnover [27]. In this sense, the contribution of LH is critical to promote continuous follicular growth, even in the presence of decreased blood LH levels [28]. Periodic surges in circulating FSH concentrations are associated with follicular wave emergence, as P4 suppresses both LH secretion and growth of the dominant follicle [26,29,30].

This stimulation in follicular growth may be dependent on the dose of P4 [31]. In our study, the serum P4 levels reached, may have allowed the number of estradiol receptors to increase in the mid–basal hypothalamus, augmenting the sensitivity to estradiol [32,33]. Therefore, such a scenario is probably caused the growth of follicles, producing more estradiol, and through positive feedback at hypothalamic level, and the enhanced GnRH release apparently generated the LH surge [34], augmenting the formation of antral preovulatory follicles, and promoting estrus activity, and subsequently ovulation [35]. Interestingly, a positive herd effect on the reproductive variables was detected, denoting the importance that management conditions may exert upon increases or reductions of the reproductive outcomes. The last response occurred even though both commercial herds were managed under intensive conditions, with quite similar nutritional and reproductive management, as well as very similar genetic make-up (dairy type), and no LW and BCS differences between herds. Although most goats in both herds ovulated, P4 dose was not related to the percentage of ovulations. This could be explained by sub–luteal serum P4 concentrations which may have been associated with abnormalities in follicular development, ovulation, oocyte health, luteal function, and fertility [6].

On the other hand, the type of breeding also influenced the reproductive outcomes; 42% of the goats exposed to NM were diagnosed pregnant, whereas only 20% goats got pregnant when subjected to AI with fresh semen. This was probably because even when using fresh semen, it could be expected a sperm decrease in both viability and motility, due to the handling and the time spent until deposited in the female´s reproductive tract, as compared to NM. Evans and Maxwell [36] reported percentages of pregnancy with AI with fresh semen greater than 40%, while we obtained less than half of that value. Another factor that could have influenced the observed low fertility in our study, is that semen experienced some degree of reduced quality due to the month when the ejaculates were obtained [37].

This study showed a clear variation in the estrous response according to the period of the anestrous season, with no influence of the P4 dose upon estrus induction in either season. While the estrus induction values favored June (transition, 100%) regarding March (deep anestrous, 70%), pregnancy rate was also higher in June, with no effect of the P4 dose. This may be because, during the time of deep anestrous, goats have a lower circulating P4 and estrogen concentration [32], while estradiol, which has basal concentrations, exerts a negative feedback at the hypothalamic level, specifically acting on the A15 dopaminergic nucleus, where it induces the synthesis and secretion of dopamine, which acts on the GnRH–producing neurons, inhibiting the frequency of synthesis and release of this hormone [28,37]. Such a situation harms the reproductive response, being stronger at the middle of the anestrous season, which could be related to the estradiol receptor population present in the hypothalamus, and a negative feedback by the gonadal steroids on the release of GnRH [38,39]. Nonetheless, even with this variability in the estrus response, our protocol was able to induce estrus in the middle of the anestrous season as well as to synchronize estrus during the reproductive transition period. On the other hand, the data obtained lead us to suppose that, even though the protocol was effective at inducing estrus response and an ovulatory rate higher than 60% in both March and June, a possible scenario could be the presence of a non-completely functional hypophyseal–pituitary–gonadal–endometrial axis, compromising the maternal recognition of pregnancy, which would increase embryo losses [40,41].

## 5. Conclusions

Results of this study confirm a multidimensional response regarding the effectiveness of P4 + eCG for the induction of estrus in anestrous goats mainly modulated by a specific time within the anestrous season (June), or even by specific management or a particular environment at herd level, although quite remarkably independent on an animal´s live weight or body condition score. While the best reproductive outcomes occurred with natural mating in June, most reproductive variables were similar either with the administration of 10 or 20 mg of exogenous natural P4 + eCG (100 IU), providing the possibility to lessen the scale of use of exogenous hormones while obtaining reasonable out of season reproductive performance. The latter result here is important not only from an animal wellbeing perspective, but also from the view of clean, green, and ethical practices.

## Figures and Tables

**Table 1:** Least square means for estrus induction (%), estrus latency (h), ovulation (%), ovulation rate (units), fertility (%), pregnancy (%), and the breeding x P4 dose interaction in adult anestrous crossbred (Saanen-Alpine-Nubian x Criollo) goats subjected to an ultra–short P4 & eCG based estrus induction protocol and breed using natural mating or artificial insemination in two commercial goat herds during the seasonal anestrous (March) in Mexico (25° N).

Response Variables		Herd	Interaction Effect ^1^ Herd × Type of Breeding × P4 Dose				P4 Dose	
			AI + 10	AI + 20	NM + 10	NM + 20	10 mg	20 mg
Estrus Induction (%)	H1	92.5 ± 0.05 ^A^	100 ± 0.08 ^a^	90 ± 0.08 ^a^	100 ± 0.08 ^a^	80 ± 0.08 ^a^	60 ± 0.05 ^B^	82.5 ± 0.05 ^A^
	H2	50 ± 0.05 ^B^	20 ± 0.08 ^b^	80 ± 0.08 ^a^	20 ± 0.08 ^b^	80 ± 0.08 ^a^		
Estrus Latency (h)	H1	49.6 ± 2.5 ^A^	46 ± 2.1 ^a^	46.6 ± 2.8 ^a^	49.4 ± 3.7 ^a^	55.5 ± 4.0 ^a^	43.5 ± 4.1 ^B^	56.3 ± 2.6 ^A^
	H2	50.2 ± 4.2 ^A^	54 ± 2.6 ^a^	60 ± 6.4 ^a^	24 ± 0.0 ^b^	63 ± 8.5 ^a^		
Ovulation (%)	H1	80 ± 0.0 ^A^	100 ± 0.14 ^a^	40 ± 0.14 ^ab^	90 ± 0.14 ^a^	70 ± 0.14 ^a^	85 ± 0.1 ^A^	70.2 ± 0.0 ^A^
	H2	75 ± 0.1 ^A^	10 ± 0.14 ^b^	60 ± 0.14 ^a^	20 ± 0.14 ^b^	60 ± 0.14 ^a^		
Ovulation rate (n)	H1	2.2 ± 0.2 ^A^	3.3 ± 0.4 ^a^	3.7 ± 0.1 ^a^	2.5 ± 0.2 ^a^	2.8 ± 0.2 ^a^	1.7 ± 0.34 ^A^	1.4 ± 0.2 ^A^
	H2	0.8 ± 0.3 ^B^	1 ± 0.0 ^b^	1.6 ± 0.1 ^ab^	1 ± 0.0 ^b^	1 ± 0.0 ^b^		
Fertility (%)	H1	42 ± 0.0 ^A^	50 ± 0.15 ^ab^	0 ± 0.0 ^b^	70 ± 0.15 ^a^	50 ± 0.15 ^ab^	42 ± 0.12 ^A^	37 ± 0.0 ^A^
	H2	37.5 ± 0.1 ^A^	0 ± 0.0 ^b^	37.5 ± 0.15 ^b^	100 ± 0.15 ^a^	50 ± 0.15 ^ab^		
Breeding Type			25 ± 0.1 ^B^		54 ± 0.1 ^A^			
Pregnancy (%)	H1	40 ± 0.0 ^A^	50 ± 0.15 ^ab^	0 ± 0.0 ^b^	70 ± 0.15 ^a^	40 ± 0.15 ^b^	32 ± 0.06 ^A^	30 ± 0.06 ^A^
	H2	22 ± 0.0 ^A^	0 ± 0.0 ^b^	30 ± 0.15 ^b^	10 ± 0.15 ^b^	50 ± 0.15 ^ab^		
Breeding Type			20 ± 0.06 ^B^		42.5 ± 0.06 ^A^			

^1^ Estrus induction, estrus latency, percentage ovulating, ovulation rate, fertility & pregnancy rate were affected (*p* < 0.05) by the Breeding × Dose interaction; ^A,B,^ Values within column (i.e., Main Effects; herds, type of breeding and progesterone dose) with different superscript, differ (*p* < 0.05); ^a, b^ Values within row (i.e., interaction effect; herd × type of breeding × progesterone dose) with different superscript, differ (*p* < 0.05).

**Table 2:** Least square means for estrus induction, estrus latency, ovulation, ovulation rate, fertility, pregnancy, and some first–order interactions in adult–anestrous crossbred (Alpine–Saanen–Nubian x Criollo) goats exposed to an ultra–short P4 & eCG based estrus induction protocol and bred either in March or June, using two P4 doses (10 or 20 mg) in two commercial herds in Mexico (25° N).

Response Variables	Herd		Interaction Effect ^1^				P4 Dose	
			Herd × Month of Breeding × P4 Dose					
			March		June			
			M10	M20	J10	J20	10 mg	20 mg
Estrus Induction (%)	H1	93.8 ± 0.04 ^A^	100 ± 0.08 ^a^	80 ± 0.08 ^a^	100 ± 0.07 ^a^	100 ± 0.07 ^a^	80.2 ± 0.04 ^A^	89.8 ± 0.04 ^A^
	H2	74.2 ± 0.05 ^B^	20 ± 0.08 ^b^	80 ± 0.08 ^a^	100 ± 0.08 ^a^	100 ± 0.08 ^a^		
Month			70.0 ± 0.05 ^B^		100 ± 0.04 ^A^			
Estrus Latency (h)	H1	51.9 ± 3.1 ^A^	49.4 ± 5.9 ^a^	44.4 ± 5.6 ^a^	59.1 ± 4.9 ^a^	57.2 ± 4.9 ^a^	42.9 ± 3.1 ^A^	52.4 ± 3.23 ^B^
	H2	42.4 ± 3.4 ^B^	4.8 ± 5.6 ^b^	50.4 ± 5.6 ^a^	57.3 ± 5.9 ^a^	58.5 ± 6.3 ^a^		
Month			37.2 ± 3.44 ^B^		58.0 ± 3.32 ^B^			
Ovulation (%)	H1	71.5 ± 0.07 ^A^	90 ± 0.14 ^a^	70 ± 0.14 ^a^	69.2 ± 0.13 ^a^	61.5 ± 0.13 ^ab^	59.3 ± 0.07 ^A^	65.7 ± 0.07 ^A^
	H2	51.5 ± 0.07 ^B^	20 ± 0.14 ^b^	60 ± 0.14 ^ab^	55.6 ± 0.15 ^ab^	75 ± 0.16 ^a^		
Ovulation rate (n)	H1	1.4 ± 0.15 ^A^	2.3 ± 0.30 ^a^	1.6 ± 0.30 ^ab^	1.1 ± 0.26 ^bc^	0.8 ± 0.26 b ^cd^	1.2 ± 0.15 ^A^	1.0 ± 0.15 ^A^
	H2	0.7 ± 0.17 ^B^	0.2 ± 0.30 ^d^	0.6 ± 0.30 ^cd^	1.1 ± 0.32 ^cb^	1.2 ± 0.34 ^bc^		
Fertility (%)	H1	61.3 ± 0.07 ^B^	100 ± 0.15 ^a^	50 ± 0.15 ^b^	100 ± 0.13 ^a^	100 ± 0.13 ^a^	55.9 ± 0.05 ^A^	56.8 ± 0.05 ^A^
	H2	100 ± 0.0 ^A^	50 ± 0.15 ^b^	62.5 ± 0.15 ^b^	100 ± 0.16 ^a^	100 ± 0.17 ^a^		
Pregnancy (%)	H1	58.1 ± 0.07 ^A^	70 ± 0.15 ^a^	40 ± 0.15 ^ab^	61.5 ± 0.13 ^a^	61.5 ± 0.13 ^a^	44 ± 0.07 ^A^	51 ± 0.07 ^A^
	H2	35.5 ± 0.08 ^B^	10 ± 0.15 ^b^	50 ± 0.15 ^ab^	33.3 ± 0.16 ^ab^	50 ± 0.17 ^ab^		

^1^ The variables estrus induction, estrus latency, percentage ovulating, ovulation rate, fertility & pregnancy rate were affected (*p* < 0.05) by the herd × month of breeding × P4 dose interaction. A,B, Values within column (Main Effects; herd, type of breeding and P4 dose) with different superscript, differ (*p* < 0.05); ^a, b, c^ Values within row (interaction effect; herd × type of breeding × P4 dose) with different superscript, differ (*p* < 0.05).

**Table 3:** Least square means for estrus induction, estrus latency ovulation, ovulation rate, fertility, pregnancy, and some three order interactions in adult–anestrous crossbred (Saanen-Alpine-Nubian x Criollo) goats exposed to an ultra–short P4 & eCG based estrus induction protocol and breed either in March or June and using two P4 doses (i.e., 10 or 20 mg) in two commercial herds in Mexico (25° N).

Interaction Effect Month of Breeding × Herd ^1^ or Month of Breeding × P4 Dose ^2^								
	March				June			
	Herd		M10	M20	Herd		J10	J20
		
	*H1*	*H2*			*H1*	*H2*		
Estrus induction (%)								
*Month × herd ^1^*	90 ± 0.06 ^a^	50 ± 0.06 ^b^			100 ± 0.05 ^a^	100 ± 0.07 ^a^		
*Month × dose ^2^*			60 ± 0.07 ^c^	80 ± 0.07 ^b^			100 ± 0.06 ^a^	100 ± 0.06 ^a^
Estrus Latency (h)								
*Month × herd ^1^*	47 ± 4.4 ^a^	27 ± 7.7 ^b^			58 ± 2.1 ^a^	58 ± 2.6 ^a^		
*Month × dose ^2^*			27 ± 4.6 ^b^	47 ± 4.6 ^a^			58.3 ± 4.4 ^a^	57.7 ± 4.5 ^a^
Ovulation (%)								
*Month × herd ^1^*	80 ± 0.10 ^a^	40 ± 0.10 ^b^			65 ± 0.09 ^ab^	64 ± 0.11 ^ab^		
Ovulation rate (n)								
*Month × herd ^1^*	1.9 ± 0.21 ^a^	0.4 ± 0.21 ^c^			1.0 ± 0.19 ^bc^	1.1 ± 0.23 ^b^		
Fertility (%)								
*Month × herd ^1^*	61.1 ± 0.11 ^a^	60 ± 0.11 ^a^			61.5 ± 0.09 ^a^	41.2 ± 0.12 ^b^		
Pregnancy (%)								
*Month × herd ^1^*	55 ± 0.11 ^ab^	30 ± 0.11 ^b^			61.5 ± 0.09 ^a^	41.10.12 ^ab^		

Italics: Denote the interaction; EI & EL were affected (*p* < 0.05) by the interactions month of breeding × herd ^1^, & the month of breeding × P4 dose ^2^.The variables EI (%), EL (h), OVP (%), OR (n), FERT (%) & PREG (%) were affected (*p* < 0.05) by the interaction month of breeding × herd ^1^. ^a, b, c^ Values within row (i.e., interaction effect; month of breeding × herd, or month of breeding x P4 dose) with different superscript differ (*p* < 0.05).

**Table 4:** Correlation coefficient matrix between live weight (LW), body conditions score (BCS), estrus induction (EI), estrus latency (EL) ovulation percentage (OVP), ovulation rate (OR), pregnancy rate (PREG) in adult anestrous crossbred (Saanen-Alpine-Nubian x Criollo) goats subjected to ultra–short progesterone & equine chorionic gonadotropin–based estrus induction protocol using different P4 doses (10 or 20 mg), bred in two different months (March or June) during the seasonal anestrous (March, 25° N) in two commercial herds in northern Mexico.

Variables	LW (kg)	BCS (units)	EL (h)	EI (%)	OVP (%)	OR (units)	PREG (%)
LW (kg)	1	0.256 0.110	0.057 0.724	0.035 0.825	0.097 0.548	0.137 0.398	−0.02 0.897
BCS (units)		1	0.172 0.288	0.346 0.028	0.446 0.003	0.581 0.001	0.201 0.217
EL (h)			1	0.836 0.001	0.581 0.001	0.440 0.004	0.421 0.006
EI (%)				1	0.801 0.001	0.640 0.001	0.562 0.001
OVP (%)					1	0.798 0.001	0.701 0.001
OR (units)						1	0.632 0.001
PREG (%)							1

## References

[1] Contreras-Villarreal V., Meza-Herrera C.A., Rivas-Muñoz R., Angel-Garcia O., Luna-Orozco J.R., Carrillo E., Mellado M., Véliz-Deras F.G. (2016). Reproductive performance of seasonally anovular mixed-bred dairy goats induced to ovulate with a combination of progesterone and eCG or estradiol. Anim. Sci. J..

[2] Martinez-Ros P., Gonzalez–Bulnes A. (2019). Efficiency of CIDR–based protocols including GnRH instead of eCG for estrus synchronization in sheep. Animals.

[3] Gonzalez-Bulnes A., Meza–Herrera C.A., Rekik M., Ben Salem H., Kridli R.T., Degenovine K.M. (2011). Limiting factors and strategies for improving reproductive outputs of small ruminants reared in semi–arid environments. Semi–Arid Environments: Agriculture, Water Supply and Vegetation.

[4] Simões J. (2015). Recent advances on synchronization of ovulation in goats, out of season, for a more sustainable production. Asian Pac. J. Reprod..

[5] Guillen-Muñoz J.M., Meza-Herrera C.A., Rivas-Muñoz R., Zuñiga-Garcia Z., Calderon-Leyva G., Mellado M., Veliz-Deras F.G. (2018). The use of female estrogenized goats as sexual stimulator of crossbred dairy males subsequently exposed to acyclic goats during two phases of the anestrous season. Theriogenology.

[6] Menchaca A., Miller V., Salveraglio V., Rubianes E. (2007). Endocrine, luteal and follicular responses after the use of the short–term protocol to synchronize ovulation in goats. Anim. Reprod. Sci..

[7] Gonzalez-Bulnes A., Veiga-Lopez A., Garcia P., Garcia-Garcia R.M., Ariznavarreta C., Sanchez M.A., Tresguerres J.A.F., Cocero J.M., Flores J.M. (2005). Effects of progestagens and prostaglandin analogues on ovarian function and embryo viability in sheep. Theriogenology.

[8] Leboeuf B., Forgerit Y., Bernelas D., Pougnard J.L., Senty E., Driancourt M.A. (2003). Efficacy of two types of vaginal sponges to control onset of oestrus, time of preovulatory LH peak and kidding rate in goats inseminated with variable numbers of spermatozoa. Theriogenology.

[9] Viñoles C., Meikle A., Martin G.B. (2009). Short–term nutritional treatments grazing legumes or feeding concentrates increase prolificacy in Corriedale ewes. Anim. Reprod. Sci..

[10] Vilariño M., Rubinaes E., Menchaca A. (2013). Ovarian response and pregnancy rate with previously used intravaginal progesterone releasing devices for fixed–time artificial insemination in sheep. Theriogenology.

[11] Martemucci G., D’Alessandro A.G. (2011). Synchronization of oestrus and ovulation by short time combined FGA, PGF2α, GnRH, eCG treatments for natural service or AI fixed–time. Anim. Reprod. Sci..

[12] Bartlewski M.P., Beard P.A., Cook J.S., Honaramooza A., Rawling C.N. (1999). Ovarian antral follicular dynamics and their relationship ëith endocrine variables throughout the oestrous cycle in breeds of sheep differing in profilificacy. J. Reprod. Fertil..

[13] Swelum A.A., Alowaimer A.N., Abouheif M.A. (2015). Use of fluorogestone acetate sponges or controlled internal drug release for estrus synchronization in ewes: Effects of hormonal profiles and reproductive performance. Theriogenology.

[14] Menchaca A., Rubianes E. (2004). New treatments associated with timed artificial insemination in small ruminants. Reprod. Fert. Develop..

[15] Rubianes E., De Castro T., Kmaid S. (1998). Estrous response after a short progesterone priming in seasonally anestrous goats. Theriogenology.

[16] Gatti M., Zunino P., Ungerfeld R. (2011). Changes in the aerobic vaginal bacterial mucous load after treatment with intravaginal sponges in anoestrous ewes: Effect of medroxiprogesterone acetate and antibiotic treatment use. Reprod. Domest. Anim..

[17] Alvarado-Espino A.S., Meza-Herrera C.A., Carrillo E., González-Álvarez V.H., Guillen-Muñoz J.M., Ángel-García O., Mellado M., Véliz–Deras F.G. (2016). Reproductive outcomes of Alpine goats primed with progesterone and treated with human chorionic gonadotropin during the anestrous–to–estrus transition season. Anim. Reprod. Sci..

[18] FASS (2010). Guide for the Care and Use of Agricultural Animals in Agricultural Research and Teaching.

[19] (2010). Guide for the Care and Use of Laboratory Animals.

[20] Gallego–Calvo L., Gatica M.C., Guzmán J.L., Zarazaga L.A. (2014). Role of body condition score and body weight in the control of seasonal reproduction in Blanca Andaluza goats. Anim. Reprod Sci..

[21] Fabre–Nys C., Gelez H. (2007). Sexual behavior in ewes and other domestic ruminants. Horm Behav..

[22] Zhao J., Wang C., Totton S.C., Cullen J.N., O’Connor A.M. (2019). Reporting and analysis of repeated measurements in preclinical animal experiments. PLoS ONE.

[23] Hauke J., Kossowki T. (2011). Comparisons of values of Pearson´s and Spearman´s correlation coefficients on the same sets of data. Quest. Geogr..

[24] Menchaca A., Rubianes E. (2002). Relation between progesterone concentrations during the early luteal phase and follicular dynamics in goats. Theriogenology.

[25] Véliz F.G., Meza-Herrera C.A., De Santiago-Miramontes M.A., Arellano-Rodriguez G., Leyva C., Rivas-Muñoz R., Mellado M. (2009). Effect of parity and progesterone priming on induction of reproductive function in Saanen goats by buck exposure. Liv. Sci..

[26] Adams G.P. (1999). Comparative patterns of follicle development and selection in ruminants. J. Reprod Fertil Suppl..

[27] Adams G.P., Matteri R.L., Ginther O.J. (1992). Effect of progesterone on ovarian follicles, emergence of follicular waves and circulating follicle–stimulating hormone in heifers. J. Reprod. Fertil..

[28] Savio J.D., Thatcher W.W., Badinga L., de la Sota R.L., Wolfenson D. (1993). Regulation of dominant follicle turnover during the oestrus cycle in cows. J. Reprod. Fertil..

[29] Meza–Herrera C.A., Berhardt L.V. (2012). Puberty, kisspeptin and glutamate: A ceaseless golden braid. Advances in Medicine and Biology.

[30] Meza–Herrera C.A., Tena–Sempere M., Astiz S., Gonzalez A. (2012). Interface between Nutrition and Reproduction: The very basis of production. Animal Reproduction in Livestock—Encyclopedia of Life Support Systems.

[31] Johnson S.K., Dailey R.A., Inskeep E.K., Lewis P.E. (1996). Effect of peripheral concentrations of progesterone on follicular growth and fertility in ewes. Dom. Anim. Endocr..

[32] Arroyo-Ledezma J., Gallegos-Sánchez J., Villa Godoy A., Valencia Méndez J. (2006). Sistemas neurales de retroalimentación durante el ciclo reproductivo anual de la oveja: Una revisión. Interciencia.

[33] Abecia J.A., Forcada F., González-Bulnes A. (2012). Hormonal control of reproduction in small ruminants. Anim. Reprod. Sci..

[34] McNeilly A.S., Picton H.M., Campbell B.K., Baird D.T. (1991). Gonadotrophic control of follicle growth in the ewe. J. Reprod. Fertil..

[35] Bartlewski P.M., Seaton P., Oliveira M.E.F., Kridli R.T., Murawski M., Schwarz T. (2016). Intrinsic determinants and predictors of superovulatory yields in sheep: Circulating concentrations of reproductive hormones, ovarian status, and antral follicular blood flow. Theriogenology.

[36] Evans G., Maxwell W. (1990). Conservación de semen durante corto tiempo. Inseminación Artificial en Ovejas y Cabras.

[37] Ángel-García O., Meza-Herrera C.A., Guillen-Muñoz J.M., Carrillo-Castellanos E., Luna-Orozco J.R., Mellado M., Véliz-Deras F.G. (2015). Seminal characteristics, libido and serum testosterone concentrations in mixed–breed goat bucks receiving testosterone during the non–breeding period. J. Appl..

[38] Arroyo-Ledezma J. (2011). Estacionalidad reproductiva de la oveja en México. Trop. Subtrop. Agroecosystems.

[39] Gonzalez-Bulnes A., Pallares P., Ovilo C. (2011). Ovulation, implantation and placentation in females with obesity and metabolic disorders: Life in the balance. Endocr. Metab. Immune Disord. Drug Targets.

[40] Meza-Herrera C.A., Ross T., Hawkins D., Hallford D. (2006). Interactions between metabolic status, pre–breeding protein supplementation, uterine pH, and embryonic mortality in ewes: Preliminary observations. Trop. Anim. Health Prod..

[41] Meza-Herrera C.A., Ross T., Hallford D., Hawkins D. (2010). Gonzalez–Bulnes, A. High periconceptional protein intake modifies uterine and embryonic relationships increasing early pregnancy losses and embryo growth retardation in sheep. Reprod. Domest. Anim..

